# Developing a capacity-building intervention for healthcare workers to improve communication skills and awareness of hard of hearing and D/deaf patients: results from a participatory action research study

**DOI:** 10.1186/s12913-024-10574-3

**Published:** 2024-03-06

**Authors:** Véronique S. Grazioli, Madison Graells, Elodie Schmutz, Odile Cantero, Tanya Sebaï, Vanessa Favre, Jessica Richème-Roos, Kevin Morisod, Michel Jeanneret, Pascal Singy, Patrick Bodenmann

**Affiliations:** 1 Department of Vulnerabilities and Social Medicine, Center for Primary Care and Public Health, Chair of Medicine for Vulnerable Populations, University of Lausanne, Lausanne, Switzerland; 2 Psychiatric Liaison Service, Lausanne University Hospital, Les Allières, Lausanne, 1011 Switzerland

**Keywords:** D/deaf, Hard of hearing, Healthcare workers, Capacity-building intervention, Communication, Participatory action research

## Abstract

**Background:**

Healthcare workers (HCWs) are commonly not prepared to properly communicate with D/deaf and hard of hearing (HoH) patients. The resulting communication challenges reinforce the existing barriers to accessing and benefiting from quality of care in these populations. In response, this study aimed to develop and evaluate a capacity-building intervention for HCWs to raise their awareness of D/deaf and HoH individuals’ experiences in healthcare and improve their capacity to communicate with these populations.

**Methods:**

This study featured a participatory action research design using qualitative and quantitative methods. The intervention was developed and tested through 4 iterative phases. Reactions (i.e., satisfaction and perception of the intervention content, quality, appropriateness and usefulness) were assessed quantitatively and qualitatively after the intervention, whereas perceived knowledge and self-efficacy in communicating with D/deaf and HoH patients and organizational payoffs (use frequency of basic rules and tools improving communication) were quantitatively assessed before, after and 6-month post-intervention.

**Results:**

Main qualitative and quantitative findings showed that the final version of the intervention reached high levels of satisfaction among participants. Next, perceived knowledge and self-efficacy scores obtained after receiving the intervention and 6 months later were significantly higher than those yielded in the initial assessment, although both scores significantly decreased at 6 months (compared to the scores obtained just after the intervention). Finally, findings showed no significant changes in organizational payoffs after receiving the intervention. Echoing these results, main qualitative findings documented that after receiving the intervention, participants felt more confident yet not more equipped to communicate with D/deaf and HoH patients.

**Conclusions:**

Findings suggest that the capacity-building intervention is a promising means to sustainably increase HCWs’ perceived knowledge and self-efficacy on how communicating with D/deaf and HoH patients, although complementary approaches and follow-up intervention reminders may be necessary to enable practice changes in the working environment.

**Supplementary Information:**

The online version contains supplementary material available at 10.1186/s12913-024-10574-3.

## Background

More than 430 million people experience disabling hearing loss worldwide and this number may reach 700 million by 2050 [1]. People with disabling hearing loss include hard of hearing (HoH) and D/deaf individuals. According to the WHO, HoH refers to individuals with hearing loss ranging from mild to severe, whereas D/deaf individuals generally experience profound hearing loss. HoH and parts of D/deaf individuals usually communicate with spoken language with support from assistive devices (e.g., hearing aids, cochlear implants), whereas most D/deaf people use Sign Language—a language *per sé—* to communicate. Importantly, D/deaf individuals communicating with Sign Language often self-identify themselves as part of a linguistic and cultural community, in which hearing loss is considered as an identity instead of a disability requiring correction (i.e., Deaf).

Access to and delivery of effective and quality healthcare among D/deaf and HoH individuals has remained a significant challenge for many years [2–7], which negatively impacts health outcomes in these populations [8]. There is, indeed, evidence that D/deaf and HoH individuals are disproportionally affected by somatic and psychological health problems compared to hearing populations [9–15]. The most significant barriers to quality healthcare stem from miscommunication issues commonly experienced by d/Deaf and HoH populations in healthcare settings [2, 16, 17]. Previous qualitative findings highlighted several communication issues’ examples, including difficulties when interacting at the desk and in waiting-rooms, conflicting representations of HoH and D/deafness between patients and healthcare workers (HCWs), communication issues during consultation and physical examination and different perspectives about how improving communication [16, 18]. Importantly, among adult Deaf from birth or early childhood, these communication issues may be reinforced by certain negative consequences of language deprivation. Language deprivation refers to the limited language acquisition opportunities in early childhood and typically results from the lack of access of visual language, such as sign language. Language deprivation can lead to negative outcomes, such as low health literacy (i.e., knowledge and skills to access, understand, process, evaluate and use health information to make decisions regarding healthcare, disease prevention and health promotion) [19–21]. There is, indeed, evidence of inadequate health literacy among Deaf adult sign language users, resulting from a limited access to health information typically tailored to hearing populations [22, 23]. Notably, the latter can pose significant communication barriers during clinical encounters with HCWs who are generally unaware of these risks [23]. As a result of these multiple encountered communication challenges, and because most often the healthcare setting is not adapted to the specific needs of D/deaf and HoH patients (e.g., providing sign language interpreting services [5]), they typically experience lack of trust and unsatisfactory patient-provider relationships [24–26] while being at risk of facing preventable adverse events [7, 27].

There is also evidence that HCWs experience their own difficulties when providing healthcare to D/deaf and HoH patients, including feeling unprepared, dissatisfied and uncomfortable [18, 26, 28, 29]. HCWs are typically not trained to properly communicate with D/deaf and HoH patients, neither are they aware of the D/deaf culture, their risks of inadequate health literacy and the importance to address them and D/deaf and HoH experiences in healthcare and specific communication needs [17, 18, 25, 30, 31]. Consequently, there have been calls to develop interventions to improve HCWs’ awareness of D/deaf and HoH experiences and communication needs [2, 6, 32].

Despite these calls, there is still a paucity of research focused on the development and implementation of such interventions [7]. Only a handful of studies conducted among pre-graduate students began to address these needs [26, 27]. A first study described an elective educational 3-hour workshop for medical students (*N* = 120) before their entry into clinical settings; the workshop included questionnaires, lectures, discussions, simulated patient’s sessions and involved D/deaf speakers. A survey completed by 41% of the students after the workshop documented high levels of satisfaction [33]. Two other studies involving 76 first-year pharmacy students and 99 first-year medical students aimed to develop role-reversal exercises in which students were the patients and D/deaf volunteers the medical staff. Findings documented that students perceived the program as educational, interesting and thought-provoking [26, 27]. Finally, a more recent study involving second-year medical students aimed to evaluate the impact of a D/deaf awareness and basic sign language training on students’ attitudes to and knowledge of D/deafness (i.e., seventeen 3-hour sessions) [34]. Findings revealed that compared to students who took another module (*N* = 30*)*, students completing the module on D/deafness (*N* = 29) had a more positive attitude to D/deaf individuals and higher knowledge scores on Deafness.

Despite these promising findings, advances remain isolated and focus on pre-graduate training exclusively. To the best of the authors’ knowledge, no previous research focused on postgraduate interventions. Further initiatives targeting broader audiences, such as postgraduate HCWs, are needed to help decrease communicational barriers encountered by D/deaf and HoH individuals in healthcare. In response, this study aimed to develop an intervention to raise HCWs’ awareness of D/deaf and HoH experience and specific needs and improve their communication skills. Specifically, the study aimed to develop and implement a capacity-building intervention among HCWs in Switzerland; to evaluate quantitatively and qualitatively participants’ satisfaction and perceptions of the intervention content, quality and appropriateness; to evaluate changes among participants on perceived knowledge and self-efficacy to communicate with the target population after receiving the intervention and 6 months later; to evaluate changes in organizational payoffs (i.e., use frequency of the skills taught during the intervention) 6 months after receiving the intervention. Considering the literature described above, we hypothesized that participants would evince higher scores on perceived knowledge, self-efficacy and organizational payoffs after receiving the intervention and 6 months later.

## Methods

### Setting

This study was conducted at the Center for Primary Care and Public Health in Lausanne (Unisanté) in collaboration with Lausanne University Hospital and several medical institutions involved with the target population in the *Canton of Vaud* (Vaud: 823’881 inhabitants, 3rd most populated Canton in Switzerland, 9.4% of Swiss inhabitants), including the primary-care ambulatory and the pharmacy units from the home institution; geriatric acute services and geriatric rehabilitation unit at Lausanne University Hospital, the local nursing home association (HévivA) and the local home support association (AVASAD) (hereafter referred as partner institutions). The project was conducted between November 2019 and December 2021. More details are provided elsewhere [35].

### Design

This was a participatory action research using qualitative and quantitative methods [36]. Consistent with the action research design, the study followed four cyclical and iterative phases: (1) *Problem identification* (identify and define the problem); (2) *Action planning* (design the action based on phase 1 data); *(3) Implementation of the action*; and (4) *Evaluation of the action* [37].

### Patient and public involvement

The study was conducted using a participatory method, equitably involving the target population, stakeholders and researchers [38]. Consistent with participatory method, the research team who implemented the research project included hearing [7], two Deaf French Sign Language users and one HoH research staff. The establishment of the research project work was discussed within the research team to ensure maximizing strengths and resources from everyone. It was decided that D/deaf and HoH research staff would be in charge of developing the capacity-building intervention, with support from the hearing research staff, and that they would provide the intervention to the participants. It was also decided that the research-related tasks (e.g., research project coordination, data collection, data analysis, draft writing) would be firstly managed by the hearing research staff. All research staff are authors of the current manuscript.

Participatory research was also operationalized through establishing an advisory committee including D/deaf and HoH individuals, association representatives and involved stakeholders (see acknowledgement section for members’ names, functions, and hearing status). Committee members provided ongoing feedback to the research team and were involved in the intervention development.

### Procedures

#### Phase 1: problem identification

This phase aimed to explore experience in healthcare, clarify the needs and gather advice on how developing the intervention among both HCWs and D/deaf and HoH individuals. Regarding HCWs, heads of partner institutions invited key staff to participate in semi-structured interviews. For D/deaf and HoH participants, recruitment was conducted with the support of D/deaf and HoH associations and within the participating nursing home. Furthermore, a video promoting study participation in French Sign Language was posted on the websites of D/deaf associations. When first getting in touch with interested participants, the research team asked them to indicate their preferred mode of communication for the interviews. When asked for, the research staff conducted the interviews with French sign language interpreters.

#### Phases 2–4: action planning, implementation and evaluation

The research team organized two half-day workshops with the committee to craft the prototype intervention. Then, the research team developed a first version of the intervention, based on qualitative findings from Phase 1 and ideas generating through workshops by the committee members. Next, the research team organized a first version of intervention (first round of testing). Heads of partner institutions invited available and interested staff to participate in the intervention. After the intervention, participants were asked to complete a questionnaire and parts of them (one per institution, randomly selected) took part in one-on-one semi-structured interview. A second improved version of the intervention was developed based on the findings of the first round of testing (second round of testing) and through discussions with the advisory committee. The resulting intervention was tested through a second round of interventions (involving new participants from the partner institutions). For the testing of the second intervention, participants were asked to complete a questionnaire at pre-intervention (T0), post-intervention (T1, just after the intervention) and at 6-month post-intervention (T2). Finally, parts of the participants (at least one per partner institution, randomly selected) took part in one-on-one semi-structured interviews at T1. Table 1 displays a summary of the measures by phases.

**Table 1:** Summary of measures by action research phase

Components	Tool	Participants	Timing of Assessment
**Phase 1: Problem Identification**			
Needs analysis	Qualitative assessment (one-on-one semi-structured interviews)	Healthcare staff D/deaf and hard of hearing individuals	Beginning of Phase 1
		Healthcare staff D/deaf and hard of hearing individuals	Beginning of Phase 1
**Phase 2: Action Planning** No measure			
**Phases 3 and 4: Action implementation and Evaluation**			
**Intervention Testing: Round 1**			
**Participants’ reactions**: perception of intervention’s content, quality and appropriateness, overall satisfaction	Adapted IMTEE questionnaire^1^ Qualitative assessment	Healthcare staff receiving intervention 1	After intervention 1
**Intervention Testing: Round 2**			
**Participants’ reactions**: perception of intervention’s content, quality and appropriateness, overall satisfaction	Adapted IMTEE questionnaire^1^ Qualitative assessment (semi-structured interviews)	Healthcare staff receiving intervention 2	T1^4^ T1
**Changes in participants**: perception of self-efficacy	Adapted IMTEE questionnaire^2^ Qualitative assessment		T0, T1, T2^4^ T1
**Organizational payoffs**: frequency of use	Adapted IMTEE questionnaire^3^ Qualitative assessment		T0, T1, T2^4^ T1

*Note.*

^1^Participants were asked to rate their satisfaction regarding the intervention and to evaluate its content, quality, and appropriateness using a 5-point Likert scale, ranging from 1 (not satisfied at all/improvement needed) to 5 (completely satisfied/very good)

^2^Participants were asked to indicate the extent to which they agree with items using a 5-point Likert-scale ranging from 1 (strongly disagree) to 6 (strongly agree)

^3^Regarding frequency of use, participants will be asked to indicate how often over the past 6 months they applied specific strategies, using a 5-point Likert scale ranging from 1 (never) to 5 (most of the time)

^4^T0=before second round of intervention testing 2; T1 = just after second round of intervention testing 2; T2 = 6 months after second round of intervention testing

### Ethics and consent to participate

This research project was deemed exempt by the Human Research Ethics Committee of Lausanne University Hospital, Vaud, Switzerland because it did not involve clinical data measurement. All procedures followed the ethical guidelines outlined in the Declaration of Helsinki. Informed consent was obtained from all participants.

Specific methods and results are presented for each phase of the research project below.

## Phase 1: Problem identification - methods

### Participants

Participants (*N* = 37) were D/deaf and HoH individuals (*n* = 19) and HCWs (*n* = 18). For HCWs, inclusion criteria included being ≥ 18 years and having cared for at least one D/deaf or a HoH patient in a healthcare work environment. A group characteristics sampling per quotas by functions (i.e., physicians, nurses, other) was selected as purposeful sampling. For D/deaf and HoH individuals, inclusion criteria included being ≥ 18 years and being d/Deaf or HoH. A group characteristics sampling per quotas by hearing loss (i.e., d/Deaf vs. HoH) was selected as purposeful sampling. We added a group characteristics sampling per quotas by age (i.e., 18–64 vs. 65 and older) for HoH individuals to account for age-related HoH (i.e., around 65) vs. more precocious forms of HoH. Interviews were conducted until reaching data saturation.

### Measures

Sociodemographic items assessed participants’ age, gender and profession. We added two items from the surveillance survey questions on hearing loss among D/deaf and HoH participants [39]. These items were used to describe the sample. Semi-structured interviews were conducted using a grid that comprised open-ended questions about participants’ experiences with healthcare and their recommendations regarding the objectives, content, and format of the intervention (see Appendixes 1 and 2). Interviews were conducted in person, by phone or by videoconference by research team members with experience in qualitative methods, with the presence of a French Sign Language interpreter when asked for. The mean duration of semi-structured interviews was 53 min.

### Data analysis plan

Semi-structured interviews were audio recorded and transcribed *verbatim*. Personal identifiable information were removed from transcripts prior to data coding. Data yielded in this first phase followed the steps of thematic analytical method [40]. Initial coding was conducted separately on a subset of data using line-by-line technique, whereby coders narrated the actions occurring in the data (allowing for inductive themes to emerge). Next, we created a codebook in consensus meetings, combing codes into themes, pooling incident-by-incident codes and removing collapsing idiosyncratic or redundant codes. Then, we used the codebook to independently double-code 10% of the sessions. After reaching adequate intercoder consistency (80%), three research members coded the remaining sessions. Feedback from the advisory committee served as a means of assessing fit and resonance of findings. This process was conducted independently with data yielded among HCWs and administrative staff and with data collected in the target population. Altlas.ti software was used to code qualitative data.

## Phase 1: Problem identification - results

### Participants and qualitative findings

In total, 37 participants took part in semi-structure interviews. The sample included HCWs from the partner institutions (*n* = 18; mean age = 36.4, *SD* = 8.6; 77.8% women; 33.3% physicians, 11.1% nurses, 16.7% administrative staff, 22.2% pharmacists, 16.7% community care assistants; 44.4% primary care ambulatory unit; 22.2% pharmacy; 22.2% geriatric acute and rehabilitation unit; 11.2% nursing home), Deaf French Sign Language users (*n* = 6), and HoH > 65 years (*n* = 5), > 65 years (*n* = 8). Among the D/deaf and HoH individuals, the average age was 50.8 years (*SD* = 20.9) and 55.6% were female. Among them, 15 (73.7%) reported using a hearing aid; when using the hearing aid, 1 reported having no difficulty hearing sounds like people voices or music, 9 reported some difficulty, 4 a lot of difficulty and 1 reported he.she cannot hear at all. Among the 4 participants using no hearing aid, 1 reported a lot of difficulty and 3 reported being unable to hear at all.

Two main themes emerged from the qualitative analysis: (1) The experience and difficulties encountered in the healthcare system with and among D/deaf and HoH patients; (2) The objectives, the form, and the content of the intervention to be developed: Opinions and recommendations. Consistent with the action research method, the first theme provides a description of the problematic in the perspective of the target populations. Main findings related to the two themes are synthetized below.

### Theme 1: the experience and difficulties encountered in the healthcare system

#### Ineffective communication and difficulties related to the attitude of professionals

Most HCWs mentioned facing difficulties when communicating with the target population. They typically mentioned a “lack of precision,” “superficial and indirect communication,” and an “inability to ensure patients’ understanding.” This echoes the comments of the D/deaf and HoH participants who reported difficulty communicating with HCWs, primarily in understanding what was being discussed.

Notably, almost all the D/deaf and HoH participants reported having experienced difficulties related to the attitude of certain HCWs. Participants perceived that HCWs “make little effort,” “quickly forget their communication needs” or that “they do not have the right reflexes to communicate with them”. For example, participant ID264 (HoH) explained: “Half of what my gynecologist says is incomprehensible, he speaks too softly, plus he has a little accent, and he doesn’t articulate.” Some participants also explained perceiving annoyance or fatigue among HCWs, with some of them refusing to adapt:

I went once to the [name of hospital], and there, there was no respect at all for D/deaf patients. It was catastrophic. I said: “I didn’t understand, I’m sorry, can we use written language?“, and it was always refused, rather curtly (ID175, D/deaf person).

Several HoH participants and one D/deaf participant noticed additional difficulties in relation to the use of the telephone. For example, participant ID122 (HoH) recalled an experience when she had to contact an ambulance for her son:

I said, ‘I’m HoH, very HoH, did he understand what that means… I don’t know! (…) he kept talking on the phone and I said: “but I’m sorry, I can’t understand you” (…). And then I just had to pray that he understood.

#### Lack of time and fear of disturbance

Almost half of the HCWs mentioned that the longer time required with a patient who is D/deaf or HoH represents a critical difficulty. Perceiving these difficulties, half of the D/deaf and HoH participants explained they are afraid to disturb HCWs, for example by asking them to repeat when they have not understood or by requiring special arrangements. A HoH participant explained: “I didn’t dare say that I didn’t understand. The lady was already stressed out” (ID61).

#### Experience

As noted by most HoH and D/deaf participants, this lack of understanding is not without consequence. Several participants expressed they felt anxious, depressed or irritated in these situations. In fact, most of HoH people and all the D/deaf participants mentioned at least one negative experience—often several, in the healthcare system healthcare:

In the operating room, the hearing aids must stay in the room, and they ask us questions with the mask on, it’s like speaking Chinese. You hear, you see the lips moving, but you understand absolutely nothing, and that’s scary (HoH, ID211).

As a result, several D/deaf and HoH participants reported apprehension about having to return to the hospital, especially when they must meet unfamiliar HCWs. However, most D/deaf and HoH participants also reported good experiences in the healthcare system, in particular when they encounter caring and supportive HCWs or when there are ways to improve communication (e.g., Sign Language interpreters).

Although less intense, HCWs mentioned negative experiences as well. Half participants explained they typically feel embarrassed or frustrated when caring a D/deaf or HoH patients because the understanding cannot be optimal.

### Theme 2: objectives, content and form of the training: recommendations

#### Raising awareness of the experiences and needs of D/deaf and HoH patients

All participants, HCWs and D/deaf or HoH confounded, mentioned that the intervention should aim at raising awareness among HCWs about D/deafness and HoH, and more specifically about the experiences and their communication needs in the healthcare system.

#### Teaching basic reflexes to improve communication and basic concepts in French sign language

Most HCWs and D/deaf and HoH participants emphasized the need to teach HCWs the basic rules for communicating with the target population, such as speaking slowly, making one’s face and mouth visible, not over-articulating or shouting, etc. Furthermore, teaching basic concepts in French Sign Language was commonly suggested by both HCWs and D/deaf and HoH participants.

#### Presenting existing tools and resources

Several HCWs recommended to include a presentation of existing tools and resources to improve communication with the target population, as well as how accessing them (e.g., French Sign Language interpreter services; cued speech coders; magnetic loop). Relatedly, some HCWs and D/deaf and HoH participants suggested creating a toolbox that could be made available after the intervention.

#### Form of the training and trainers

According to most participants, the intervention should last about half a day, although some participants felt that a more extensive training would be necessary. Regarding the form, most participants mentioned that the training should include practical components, such as role-playing. Importantly, most participants felt that the training should be delivered by D/deaf and/or HoH trainers.

## Phase 2: Action planning - round 1 of testing

**Fig. 1:**
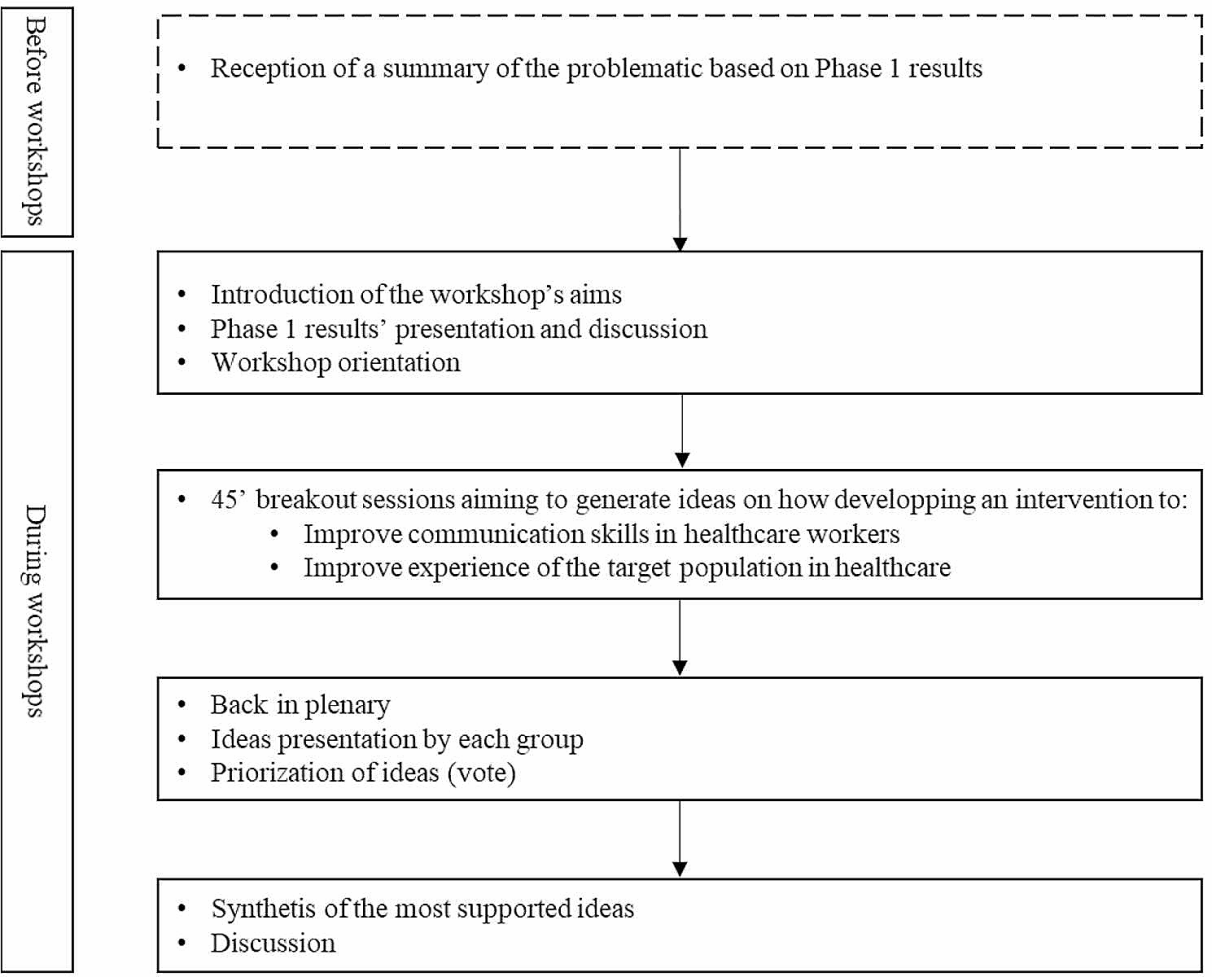
Workshop process with members of the advisory committee

Two 2.5-hour workshops were conducted with the advisory committee to develop ideas on the form and content of the intervention to be developed based on the results of Phase 1. The workshops were facilitated by the first author and 2 members of the research team, with French Sign Language interpreters. In total, 11 members participated in these workshops. Fig. 1 describes workshops’ process.

Main findings from these workshops indicated that the intervention should include 3 parts addressing 3 objectives:

1. Raising awareness of the experiences and communication needs of D/deaf and HoH patients.
2. Raising awareness of the importance of the first contact and teaching good practices to reassure D/deaf and HoH patients to promote a good follow-up.
3. Introducing existing tools and basic rules for improving communication with D/deaf and HoH patients.

## Phase 3: Action implementation - round 1 of testing

The research team developed and organized the intervention according to results from phase 1 and recommendations from the advisory committee during the workshops (phase 2). It was decided that the intervention would take place over half a day and that it would include theoretical parts, testimonies, situational and practical exercises. In addition, the research team decided to use existing videos and create new ones to increase awareness of the target population’s experiences and reinforce the learning of good practices to improve communication. Finally, it was decided that the introduction of existing tools and good practices to improve communication would be achieved through the development of a toolbox that would be sent to participants prior to the training and introduced during the intervention. Table 2 describes the content and the different parts of the intervention developed to meet each objective.

**Table 2:** Content of the intervention by objective

**Objective 1: To understand the experiences and communication needs of people who are D/deaf or hard of hearing**	
Content	Form
Deaf History and Culture	Theoretical presentation
Deafness and hearing loss: • Definition • Hearing loss degrees and causes • Prevalence • Consequences (e.g., local language learning; isolation; inadequate health literacy)	Theoretical presentation Trainers’ testimonies
Awareness of the experiences of D/deaf and HoH patients in healthcare setting	Broadcasting of 4 films displaying testimonies of negative experiences in healthcare of a HoH and a Deaf patient.
Diversity of means and needs of communication • Sign language • Cued Speech • Communication supports (e.g., sign language interpreter; cued speech coder; hearing assistive devices, magnetic loop, lip reading) • Diversity of needs (e.g., depending on communication mode, local language proficiency and education, hearing loss degree) • Clinical encounter with a sign language interpreter (and intermediator) • Clinical encounter with a cued speech coder	Theoretical presentation Link to toolbox (part 3)
Reminder of the legal basis	Theoretical presentation
Role-playing situations	Situation with limited hearing (using earplugs and lowering the volume of the computer)
**Objective 2: To understand the importance of the first contact and apply good practices to reassure patients who are D/deaf or hard of hearing and to promote good follow-up care**	
Good practices (desk, waiting room and clinical encounter) • Make an appointment (e.g., smart phone messages, email, online sign language interpreting services) • Reception attitude and ground rules for better communication • Identification of communication’s needs (e.g., sign language interpreter, cued speech coder, magnetic loop) • How to behave in the waiting room and during the clinical encounter	Theoretical presentation• Broadcast of 3 educational films reinforcing good practices • Reception • Waiting room • Consultation
Rudimentary knowledge of French sign language	Theoretical presentation Practice (learning some basic words)
Introduction to lip reading	Theoretical presentation Putting it into practice
**Objective 3: To know the different tools available and the basic rules for communication and how and when to use them: Toolbox**	
Contents of the toolbox	• Course material of the intervention (condensed version). • Visual documents reminding good practices. • Visual document to be used with D/deaf and HoH patients for the choice of communication means. • Documents explaining the different interpreting possibilities. • Links and contacts of professionals in cued speech and French Sign Language. • Documentation on the magnetic loop. • Link to an Internet site offering a lexicon in French Sign Language. • Pictogrammes. • Links of the existing training possibilities (French Sign Language, cued speech, lip reading) • Summary document of the different degrees of hearing loss.
	Toolkit sent to participants before the intervention. Presented and discussed throughout the intervention.

A first test of the intervention was carried out in April 2021 with 13 HCWs from the partner institutions. Due to the COVID-19 pandemic, the intervention was carried out by videoconference. The half-day intervention was given by the 3 members of the research team who are themselves Deaf and HoH, with logistical support from the remaining research team. Consistent with research staff communication modes, the two Deaf research staff provided the intervention in French Sign Language, whereas the HoH research staff communicated in French during the intervention. The intervention was therefore conducted with the presence of 2 interpreters in French Sign Language.

## Phase 4: Action evaluation - round 1 of testing: methods

### Measures

The *Integrated Model of Training Evaluation and Effectiveness* (IMTEE) [41] guided the intervention evaluation [36]. A quantitative questionnaire was developed to fit intervention content and adapted from past research using the IMTEE framework [42, 43]. Once developed, it was pre-tested in 10 HCWs to ensure items’ understanding. For the first round of testing, the questionnaire assessed reactions of participants regarding the intervention (i.e., satisfaction and perception of the intervention content, quality, appropriateness, and usefulness) after receiving the intervention. The quantitative evaluation was completed by a qualitative assessment exploring the same dimensions through one-on-one semi-structured interviews conducted by videoconference with an interview grid (see Appendix 3).

### Quantitative and qualitative analysis

Descriptive statistics were used to summarize quantitative data. Similar to Phase 1, semi-structured interviews were conducted until reaching data saturation, audio-recorded and transcribed *verbatim* by research assistants. Qualitative assessment was conducted based on findings from phase 1 and aimed to complement quantitative findings. Hence, we used a deductive thematic analysis consisting of analyzing data according to an existing framework (i.e., the themes of the interview grid) [44].

## Phase 4: Action evaluation - round 1 of testing: results

### Quantitative results

#### Participants

A total of 13 participants took part in the intervention (76.9% women; average age = 41.3 years, *SD =* 12.1). Among the participants, 23.1% were nurses, 30.8% pharmacists, 15.4% administrative staff and 30.8% others (dietician, community care assistant, health auxiliary; 30.8% pharmacy unit; 23% primary-care ambulatory unit; 15.4% nursing home; 15.4% acute and rehabilitation geriatric unit; 15.4% home-support association). All participants completed the questionnaire.

#### Results

Table 3 presents the descriptive statistics from the questionnaires. Over 90% agreed or strongly agreed that the intervention met their expectations and needs in the field. Next, most participants agreed or strongly agreed that the intervention would influence their practice, that it strengthened their understanding of the target population’s experiences and specific needs, and that they knew the basic principles to apply to improve communication with these patients. However, more than 30% of the participants indicated that they felt only moderately equipped to receive the target population after the intervention. All parts of the intervention were judged as good or very good by most participants except for the part on the history of the Deaf, which was evaluated as average by most participants.

**Table 3:** Descriptive statistics of participants’ reaction regarding the intervention (first round of testing; *N* = 13)

Questions					
**General Assessment**	1 (%)	2 (%)	3 (%)	4 (%)	5 (%)
In general, how do you evaluate the training?^1^	0	0	0	30.8	69.2
The training met my expectations^2^	0	0	8.3	33.3	58.3
The training met my needs in the field^2^	0	7.7	7.7	46.2	38.5
**Content**					
In general, how would you rate the content of the training?^1^	0	0	0	53.8	46.2
*How would you rate the following parts*^*1*^:					
History of the Deaf	0	0	58.3	33.3	8.23
Deafness and hard of hearing	0	0	0	38.5	61.5
The Deaf Community and Culture	0	0	7.7	69.2	23.1
Communication needs and tools	0	0	7.7	23.1	69.2
Good practices	0	0	0	23.1	76.9
Introduction of French Sign Language	0	0	17.7	41.7	41.7
Introduction to Lip reading	0	0	0	38.5	61.5
The toolbox	0	0	0	30.8	69.2
The content of the training was sufficient^2^	0	0	23.1	38.5	38.5
The content of the training is applicable to my workplace^2^	0	0	16.7	25	58.3
The content of the training is useful for my practice^2^	0	0	15.4	15.4	69.2
**Organization and logistics**					
How would you rate the organization of the training?^1^	0	0	0	53.8	46.2
What do you think of the intervention’s support (slides)?^1^	0	0	7.7	61.5	30.8
What do you think of the videos?^1^	0	0	0	15.4	84.6
What do you think of the duration of the training^3^	0	7.7	69.2	15.4	7.7
**Usefulness**					
The training will influence my practice^2^	0	0	7.7	46.2	46.2
The training reinforced/improved my understanding of the experiences of people who are D/deaf and hard of hearing and their specific communication needs^2^	0	0	0	69.2	30.8
The training gave me a better understanding of the basic principles to apply in order to improve communication with people who are D/deaf or hard of hearing^2^	0	0	0	41.7	58.3
The training allowed me to learn more about the tools available to improve communication with D/ deaf or HoH patients^2^	0	0	7.7	38.5	53.8
After having followed the training and with the toolbox, I am able to pass on to my colleagues which basic principles to apply and which tools to use to improve communication with D/deaf or HoH patients^2^	0	0	15.4	46.2	38.5
At the end of the training and with the toolbox, I feel equipped to receive peopled/deaf or HoH patients^2^	0	0	30.8	53.8	15.4

*Rating.*^1^ 1 = very bad, 2 = bad, 3 = average, 4 = good, 5 = very good;^2^ 1 = disagree, 2 = disagree, 3 = somewhat agree, 4 = agree, 5 = strongly agree;^3^ 1 = really too short, 2 = too short, 3 = good, 4 = too long, 5 = really too long

### Qualitative results

#### Participants

Six participants took part in a semi-structured individual interview (83% women; 33.3% community care assistants, 16.7% pharmacists, 16.7% receptionists, 33.3% nurses). Findings yielded from the analysis are synthetized below. The mean duration of the interviews was 51 min.

#### Overall impression of the intervention

The intervention was consistently well perceived, often described as “interesting” and “rich.” The most appreciated aspects of the intervention included learning about the experiences of D/deaf and HoH people and their different communication needs, the testimonies and exchanges with the trainers, and the fact that they were themselves Deaf and HoH: “Having… interlocutors… from the… hearing impaired community, it was very enriching; we felt that their experience went with the theory, it was really an added value” (ID 2). Finally, most participants mentioned appreciating the balance between theoretical and practical moments as well as the videos often perceived as reinforcing the learning.

#### Perceptions of the usefulness of the intervention

Most participants mentioned that the intervention was useful and that they intended to integrate the elements taught into their practice. Furthermore, participants commonly reported that they felt equipped to pass on certain elements acquired to their colleagues, even though some noticed that it could not replace the intervention itself, as participation was considered essential to raise awareness of the experiences of the target population. However, two participants reported feeling not more equipped to communicate with HoH elderly after receiving the intervention:

I work in nursing home, unfortunately following this training, I won’t really have the tools. When you have elderly people who suffer from hearing loss, it’s true that, despite what we’ve seen, we still don’t really manage to communicate (ID 1).

#### Suggested improvements related to the content of the intervention

All participants felt that the section on Deaf history was too long, whereas the length of the part related to the good practices was often perceived as too short. Participants commonly suggested to add role playing to reinforce this part. Regarding the introduction of the French Sign Language, half participants reported that they were unable to retain the words introduced and suggested either deleting this part or reducing the number of words to ensure learning. Finally, two participants suggested to add a section focusing on HoH elderly.

#### Suggested improvements related to the form of the intervention

Although the videoconference intervention was perceived as well organized and functional, most participants would have preferred to participate in person to favor interaction. The duration of the intervention was considered too short by half of the participants, who suggested that it should be given over a full day to increase the practical parts and reinforce the learning. According to the other participants, the intervention length was adapted (2 participants) or too long (1 participant).

## Back to phase 2: Action planning - round 2 of testing

The next step was to adjust the intervention based on the results from the previous phase with input from the advisory committee, leading to the finale version of the intervention. This phase began with working sessions with the advisory committee, during which the research team presented the content and format of the intervention tested in the previous phase as well as the results from the evaluation. These sessions aimed to collect committee member’s feedback on possible improvements to be made and were conducted with French Sign Language interpreters. Committee members recommended to clarify the differences between sign language interpreter, community interpreter and intermediary interpreter. They also suggested to shorten the theory, lengthen the practical parts, and add an introduction on cued speech as well as a section on communication with HoH elderly people. Finally, regarding the toolbox, they recommended to add pictures illustrating how to sign basic words in French Sign Language as well as a document clarifying the functioning of the remote interpreting.

## Back to phase 3: action implementation - round 2 of testing

### The finale intervention

According to both results from the evaluation and the advisory committee recommendations, the following modifications were made:

1. Less theory, more practice: Synthesis of the theoretical parts, focus on good practices, and addition of role playing. Reinforcement of the toolbox usefulness during the intervention.
2. HoH elderly: Addition of a section dedicated on how to promote good communication with this population (theoretical part and role playing).
3. Sign Language interpretation: Addition of a slide clarifying the interpretation process and the distinctions between interpreter, community interpreter and intermediary interpreter; addition of a document clarifying these roles and the functioning of the remote interpreting in the toolbox.
4. Cued Speech: Addition of a practical introduction and cued-speech-related information’s in the toolbox.
5. French Sign Language introduction: Aim to teach 4 words (instead of 10) and addition (in the toolbox) of videos teaching how to interpret a few basic words in French Sign Language to welcome a Deaf patient.

The duration of the intervention was kept to half a day and the second version was organized face-to-face. It was given twice, in June 2021, to a total of 28 participants from the project’s partner institutions who had not participated in the first test of the intervention.

## Back to phase 4: Action evaluation - round 2 of testing: methods

### Measures

Consistent with the *Integrated Model of Training Evaluation and Effectiveness* (IMTEE) [41] the quantitative questionnaire assessed the following dimensions:

#### Intervention content and design

 Seven items assessed reactions of participants regarding the intervention after receiving the intervention (T1).

#### Changes in participants

Eight items measured perceived self-efficacy to apply the skills taught during the intervention (α = 77); 10 items assessed perceived acquisition of knowledge (α = 88*)* [42, 43]. These dimensions were assessed at T0, T1 and at T2 and were used as dependent variables in the main analysis.

#### Organizational payoffs

Four items measured use frequency of the newly acquired skills (α = 94) [42, 43]. A total score was computed, hereafter referred as “organizational payoffs”. This dimension was assessed at T1 and T2 and was used as a dependent variable.

The quantitative evaluation was completed by a qualitative assessment exploring the same dimensions through one-on-one semi-structured interviews conducted by videoconference (see Appendix 4).

### Quantitative and qualitative analysis

Quantitative data were screened for missing cases, outliers, and normality of distributions using descriptive statistics. Population-averaged estimating equation (GEE) modeling were used to test time as a predictor of the dependent variables (i.e., self-efficacy, knowledge and organizational payoffs) over the 6-month follow-up period [45]. GEE are marginal models that can take into account nonindependence resulting from data clustering (e.g., longitudinal data). As the three dependent variables were normally distributed, we specified the Gaussian distribution. All models were adjusted for professions, age and gender. Analyses were conducted using STATA. The significance level was set at *p* =.05. Qualitative analysis mirrored the methods used in the first round of testing.

## Back to phase 4: action evaluation - round 2 of testing: results

### Quantitative results

#### Participants

In total, 28 HCWs from the partner institutions participated in the second round of testing. The mean age was 43.61 (*SD* = 11.47) and the sample included predominantly female participants (96.4% women; 35.1% nurses, 17.9% community healthcare assistants, health auxiliaries; 21.4% pharmacists, pharmacy assistants; 21.4% administrative staff; 3.6% other; 57.1% primary-care ambulatory unit; 21.5% pharmacy unit; 14.3% nursing home; 7.1% home support unit).

#### Attrition analysis

All participants (*N* = 28) completed the questionnaires at T0 and T1; at T2, 25 participants (89.3%) completed the questionnaire. We conducted attrition tests comparing participants who completed the questionnaire at T2 with those who did not to verify whether the data were missing at random, which revealed no significant differences in gender, age and professions.

#### Participants’ reactions regarding the intervention content and design

As shown in Table 4, all participants rated the training as good or very good and agreed or strongly agreed that it met their expectations. According to most of them, the training met the needs of the field and was applicable and useful for practice.

**Table 4:** Descriptive statistics of participants’ reactions regarding the intervention content and design (second round of testing; *N* = 28)

General assessment					
Questions	1 (%)	2 (%)	3 (%)	4 (%)	5 (%)
In general, how do you evaluate the training?^1^	0	0	0	21.4	78.6
The training met my expectations^2^	0	0	0	29.6	70.4
The training met my needs in the field^2^	0	0	11.1	33.3	55.6
**Content**					
The content of the training is applicable to my workplace^2^	0	0	10.7	50	39.3
The content is useful for my practice	0	0	14.3	32.1	53.6
**Logistics**					
How would you rate the organization of the training?^1^	0	0	0	28.6	71.4
What do you think of the duration of the training?^3^	0	7.4	44.4	29.6	18.5

*Rating.*^1^ 1 = very bad, 2 = bad, 3 = average, 4 = good, 5 = very good;^2^ 1 = disagree, 2 = disagree, 3 = somewhat agree, 4 = agree, 5 = strongly agree;^3^ 1 = really too short, 2 = too short, 3 = good, 4 = too long, 5 = really too long

#### Changes in participants

Table 5 displays descriptive statistics of items assessing participants’ perceived knowledge and self-efficacy at T0, T1 and T2 as well as organizational payoffs at T1 and T2. Table 6 presents GEE model statistics and parameter estimates. Both models testing the associations of time with knowledge and self-efficacy were significant. Findings showed that associations of time with knowledge and self-efficacy were significant, such that knowledge and self-efficacy increased over time. In contrast, the model and the association of time with institutional payoffs were not significant, indicating that institutional payoffs did not change over time.

**Table 5:** Descriptive statistics of participants’ perceived knowledge and self-efficacy at T0, T1 and T2 and organizational payoffs at T1 and T2 (second round of testing; *N* = 28)

	**T0**	**T1**	**T2**
**Knowledge** ^**1**^	**Mean (** ***SD*** **)**		
1. People who are D/deaf or HoH encounter difficulties when interacting with healthcare and administrative staff	4 (0.9)	4.43 (0.63)	4.04 (0.96)
2. There is a Deaf community that has its own culture	3.99 (1.17)	4.78 (0.42)	4.38 (1.14)
3. I know the difference between deafness and hard of hearing	3.29 (1.05)	4.54 (0.64)	4.13 (0.68)
4. I know the consequences of deafness on the learning of spoken and written language	2.89 (1.26)	4.36 (0.73)	3.91 (0.52)
5. I know how French Sign Language and cued speech language are different	2.11 (1.19)	4.71 (0.66)	4.09 (0.67)
6. I know what tools I can use to improve communication with patients who are D/deaf or HoH	2.68 (0.95)	4.43 (0.57)	3.96 (0.56)
7. I can distinguish between a French Sign Language interpreter, an intermediary and a cued speech coder	1.71 (1.11)	4.5 (0.64)	3.71 (0.75)
8. I know what lip reading is	3.29 (1.11)	4.58 (0.36)	4.2 (0.62)
**Knowledge total score**	**23.41 (5.35)**	**36.58 (3.16)**	**32.5 (3.5)**
**Self-efficacy** ^**1**^			
1. I know how to contact a D/ deaf or HoH patient for an appointment	2.43 (1.26)	4.32 (0.77)	3.64 (0.76)
2. I know what basic reflexes to adopt to improve communication with a D/deaf or HoH patient	3.18 (0.95)	4.5 (0.64)	4.08 (0.49)
3. I know how to identify the communication needs of D/deaf or HoH patient who come to my workplace	2.89 (0.96)	4.29 (0.66)	3.84 (0.62)
4. I can distinguish when to use an interpreter in French Sign Language, an intermediary or a cued speech coder	1.96 (1.04)	3.93 (0.81)	3.32 (0.9)
5. I know how to contact an interpreter in French Sign Language, an intermediary or a cued speech coder	1.93 (1.22)	3.93 (0.83)	3.24 (0.97)
6. I know how to behave when I pick up a D/deaf or HoH patient in the waiting room	2.89 (1.03)	4.61 (0.63)	4.17 (0.64)
7. I know strategies to improve the interactions of an older patient with a hearing loss in their daily life	2.43 (1.34)	4.39 (0.57)	3.92 (0.72)
8. I can say 2 greeting words in French Sign Language	1.68 (1.19)	4.39 (0.83)	3.56 (1.16)
9. I can code 2 greeting words in Complete Spoken Language	1.54 (0.84)	3.26 (1.1)	2.12 (1.04)
10. I know how to make lip-reading easier for my interlocutor	2.36 (1.13)	4.5 (0.75)	3.96 (0.68)
**Self-efficacy total score**	**23.29 (7.62)**	**42.17 (5.7)**	**35.88 (5.34)**
		**T1**	**T2**
**Institutional payoffs** ^**2**^	**Mean (** ***SD*** **)**		
*In the past 6 months, when you met with people who were deaf or hard of hearing, how often did you...*			
1. Applied basic reflexes to improve communication with D/deaf or HoH patients?		3.08 (1.12)	2.8 (1.35)
2. Used communication aids to improve interactions with D/deaf or HoH patients?		2.96 (1.2)	2.56 (1.29)
3. Reminded any of your colleagues of the basic reflexes to adopt to improve communication with D/deaf or HoH patients?		2.32 (1.28)	2.2 (1.19)
4. Reminded any of your colleagues what tools are available to improve communication with patients D/deaf or HoH patients?		2.24 (1.3)	2.36 (1.19)
**Institutional payoffs total score**		10.77 (4.54))	10.18 (4.54)

**Table 6:** GEE Model Effects and Parameters Showing the Associations of Time with Knowledge, Self-efficacy and Organizational Payoffs (*N* = 28)

Variables	Knowledge		Self-efficacy		Organizational Payoffs	
	Wald χ2	*B(SE)*	Wald χ2	*B(SE)*	Wald χ2	*B(SE)*
Model	38.9***		30.82***		2.58	
Time		4.79(0.79)***		6.56(1.19)***		− 0.65(1.22)
Profession		− 0.27(0.51)		0.19(0.78)		0.6(0.5)
Gender		0.44(3.36)		0.89(0.09)		-2.32(3.11)
Age		− 0.07(0.06)		− 0.0(0.09)		− 0.05(0.05)

Note. ^*^= ^***^=*p* <.001;

Additional paired sample t-tests were conducted to compare knowledge and self-efficacy scores between T0 and T1, T0 and T2 and T1 and T2. Regarding knowledge, results indicated a significant increase in scores between T0 (*M* = 23.41, *SD* = 5.35) and T1 (*M* = 36.48, *SD* = 3.16), *t* (27) = -12.32, *p* <.001. The eta squared statistic (0.84) indicates a large effect size. The results further showed a significant decrease in scores between T1 (*M* = 36.88, *SD* = 2.35) and T2 (*M* = 32.5, *SD* = 3.5), *t* (23) = 5.84, *p* <.001. The eta squared statistic (0.59) indicates a large effect size. Although scores decreased significantly over time, the results indicated that scores at T2 (*M* = 32.5, *SD* = 3.5) were significantly higher than those at T0 (*M* = 23.41, *SD* = 5.46), *t* (23) = -7.81, *p* <.001. The eta squared statistic (0.73) indicates a large effect size.

Regarding self-efficacy, the results indicated a significant increase in scores between T0 (*M* = 23.29, *SD* = 7.62) and T1 (*M* = 42.17, *SD* = 5.77), *t* (27) = -12.32, *p* <.001. The eta squared statistic (0.84) indicates a large effect size. The results also showed a significant decrease in scores between T1 (*M* = 42.15, *SD* = 6.08) and T2 (*M* = 35.88, *SD* = 5.31), *t* (24) = 4.9, *p* <.001. The eta squared statistic (0.50) indicated a large effect size. Finally, the results indicated that the scores obtained at T2 (*M* = 35.88, *SD* = 5.34) were significantly higher than those obtained at T0 (*M* = 23.63, *SD* = 7.89), *t* (24) = -10.23, *p* <.001. The eta squared statistic (0.80) indicates a large effect size.

### Qualitative results

#### Participants

Ten participants took part in a semi-structured individual interview (90% women; 20% pharmacists; 20% community care assistants, 20% receptionists, 40% nurses). Findings yielded from the analysis are synthetized below. The mean duration of the interviews was 42 min.

#### The intervention is well received and raises awareness of the experience and communication needs of D/deaf and HoH patients

##### Positive perceptions of the intervention.

The intervention was systematically well perceived by the participants who described it as “interesting,” “fascinating,” “instructive,” “useful” and “rewarding.” In line with findings from round 1, according to all participants, the trainers, and in particular the fact that they were themselves Deaf and HoH was the main added value of the intervention:

The way it is given is really the best of the training. Having HoH or Deaf people, who are there in front of us with interpreters who do it live (…) that really allows us to have an understanding a little more than just theoretical (…). Because, if you want to talk to them, you have to… well, you have to find ways to raise your hand, take off your mask, speak before you get a translation (ID 4).

The fact that the trainers talked about their life experiences was pointed as a crucial ingredient of the intervention. Participant 3 disclosed for instance: “The strength of the training was precisely that… they dared to talk about their feelings and their experiences; those were the parts that touched the most.”

#### The intervention raises awareness of experience and communication needs of D/deaf and HoH patients

All participants perceived that the intervention raised awareness of experience and communication needs of D/deaf and HoH patients. According to participants, several ingredients enabled to reach this aim, including the fact that the trainers were Deaf and HoH themselves. Other cited ingredients included role-playing, situational exercises, and educational videos. For instance, referring to situational exercise during which participants were required to wear headphone mute to decrease hearing capacity, participant 73 reported: “To put yourself in their shoes through the indispositions of… of the headphone mute and to say to yourself well I have a tiny glimpse of what they can live with every day.”

#### The intervention improves perceived knowledge of how communicating with D/deaf and HoH patients

Participants commonly explained that they improved their knowledge of the existing tools and basic reflexes to improve communication with D/deaf and HoH patients:

The fact of knowing how to look face to face, speak nicely, with gestures. After writing too, there are also videoconferences with [sign language] interpreters… There are certain telephone numbers, hotlines (…). There are many easy solutions now (ID 6).

Furthermore, several participants explained that they had never heard of cued speech and magnetic loops before, whereas others explained that they improved their understanding of the differences between Deaf, deaf and HoH. Finally, participants commonly reported they learnt the extent to which communication needs are diverse across D/deaf and HoH patients.

#### The perceived anticipated benefits of the intervention in the working environment

##### Feeling more self-confident and more at ease.

Most participants explained that the intervention provided them with a basis enabling to feel more self-confident and more at ease with D/deaf and HoH patients in the working environment. Participant 47 disclosed for instance: “I wouldn’t say I know everything, but perhaps compared to a colleague who didn’t attend the training, I would be at ease.” Consequently, participants commonly explained that after the intervention they would be “less shy” with D/deaf and HoH and they would dare trying different means to communicate with them. According to participant 73, engaging with the target population was considered a priority: “This is the main tool, you must not be shy, you must try to mime, no matter how and show that you want to communicate.”

##### Feeling somewhat more equipped to welcome and reassure D/deaf and HoH patients.

Participants commonly reported they felt more equipped to receive and reassure D/deaf and HoH patients after attending the intervention:

Yeah, I feel more equipped. (…) Now I think I’m a little bit quicker and I’m thinking: ah yes! I have this toolbox. In addition, we are lucky in that we know in advance the patients we have (…). So I can prepare myself by opening the toolbox. And even in front of him [a D/deaf or HoH patient], I think I’ll still remember it pretty well (ID 3).

Relatedly, participants often mentioned that they would make more efforts in the interaction with D/deaf and HoH patients after attending the intervention. Participant 96 reflected for instance:

I was a bit passive. I was waiting for her to tell me what to do. And if she didn’t say anything… well… I stayed in my corner too (…). But I think that today, I would go more into the interaction. I would suggest tools, methods or… yes, quite simply, writing.

When asked to reflect on concrete changes they anticipate making in the working environment, participants commonly evoked the use of different communication tools introduced during the intervention. Participant 47 noticed for instance that she would “really use mimicry more,” that “she would lift the mask, try to articulate.” Other participants commonly evoked that they would try diverse means to communicate: “Maybe it will be drawings. The small cards. And to mime (…). Afterwards it will be to go and look for a [sign language] interpreter. (…) We can write (…). Yeah, no there are quite a few tools.” (ID 96).

Corollary, participants often explained that they would start the interaction with D/deaf and HoH patients by identifying their own specific communication needs:

Before starting a conversation, (…) I would ask in some ways what wants to use the person. If he prefers to write… that’s it. Or if they need a [sign language] interpreter actually over the phone… that would be a step that I would change now (ID 70).

#### Feeling more at ease yet not more equipped to communication with D/deaf and HoH patients

Despite the perceived benefits described above, participants commonly nuanced the anticipated changes in the working environment.

##### Too much theory and not enough practice to consolidate learning and feel equipped.

The intervention was often perceived as too theoretical. Participant 21 mentioned for instance: “Yes, on the theory level we got a lot of stuff. But I think I’m still as lost if I have someone in front of me.” The intervention length and lack of practice were commonly perceived as preventing participants from consolidating learning and thereby feeling more equipped:

I would have found very interesting to do more practice. We did some, and I was able to participate in a practical case. And it’s true that hearing how to react is easy. But afterwards, when you find yourself in the situation, suddenly, you don’t know what to do when you’ve just heard the theory 3 seconds before (ID 8).

##### In its format, the toolbox is difficult to use in the field.

Some participants questioned the toolbox usefulness. Its format was perceived as suboptimal and the fact that it was provided on the USB key was perceived as unpractical.

#### Recommendations to improve the intervention

Participants suggested several ways to improve the intervention and favor learning consolidation over time, such as including more role playing. Relatedly, a few participants suggested to organize the intervention in two half days, providing additional time for practical exercises. Furthermore, some participants evoked the importance of repetition to consolidate learning. The idea to organize regular reminders in staff meetings to avoid forgetting over time was suggested by some of the participants, whereas others recommended to propose an intervention follow-up or to have a resource person within the institution. For some participants, this follow-up training would aim to teach French Sign Language, whereas others suggested to receive coaching directly in the field after the initial intervention. Finally, participants commonly recommended to improve the toolbox format, making it more practical and user-friendly.

## Discussion

This study aimed to develop and evaluate a capacity-building intervention aimed at raising HCWs’ D/deaf and HoH experience and specific needs in healthcare and improve their communication skills. Main findings revealed that perceived knowledge related to D/deafness and HoH and existing tools to improve communication and perceived self-efficacy regarding how to communicate with D/deaf and HoH patients significantly improved after receiving the final version of the intervention, whereas there were no significant changes in organizational payoffs in the working environment (i.e., use frequency of basic reflexes and communication tools). These quantitative findings were corroborated by qualitative results documenting that participants typically felt more self-confident yet not necessary more equipped to receive and interact with D/deaf and HoH patients after the intervention.

### Co-developing interventions to improve communication with D/deaf and HoH patients together with members of the target populations meet needs in healthcare setting

Consistent with research action method, the study started with a qualitative exploration aiming to describe the problematic from the perspective of involved populations and gather recommendations regarding the intervention’s content and form. In line with past research [2, 16, 17, 46], findings documented that D/deaf, HoH patients and HCWs face individual and structural difficulties severely impeding communication and leading to negative experiences in healthcare settings. The latter were described as sustaining an often already-existing apprehension to seek care and ultimately reinforcing barriers in the target population. These findings confirm the relevance to develop and disseminate interventions aimed at improving HCWs’ communication skills and ultimately help decrease barriers to healthcare among D/deaf and HoH individuals.

Consistent with existing literature focused on pre-graduate training [32–34, 47], findings revealed that the intervention should aim at raising awareness of the experiences and diverse communication needs of the target population, teach the basic communication rules to improve communication, introduce the French Sign Language, the cued speech and the resources and existing tools to support communication (e.g., French sign Language interpreter and cued speech coders services, magnetic loop). Furthermore, participants consistently advised to involve D/deaf and HoH in the intervention development and delivery. This recommendation is consistent with past studies testing interventions among medical and pharmacy students that typically involved the target populations [33, 48]. Consistent with the community-based participatory research paradigm [49], involving members of the target populations in the development and delivery of the intervention is critical to ensure embracing insights and perspectives of involved people and ultimately develop an intervention tailored to meet the specific needs encountered in the field. Importantly, our qualitative findings among participants who received the intervention highlighted that having the intervention delivered by Deaf and HoH trainers was perceived as the added value and the main ingredient of the intervention effectiveness. Taken together, our findings coupled with past research suggest that future efforts aiming to develop and disseminate such interventions should systematically involve D/deaf and HoH individuals.

### The intervention is promising to improve HCWs’ perceived knowledge and self-confidence in communicating with D/deaf and HoH patients but additional components are needed to ensure sustainable changes in the working environment

Consistent with the few previous studies conducted among pre-graduate students [33, 48, 50], our results showed high levels of satisfaction among the participants who attended one of the interventions provided in this research. These results attest to the interest of the HCWs in this problematic and are reinforced by the fact that the intervention was given entirely by D/deaf and HoH trainers. As mentioned earlier, this aspect was percived as the added value during the qualitative evaluations of the interventions.

As developed in this study, the capacity-building intervention aimed to have participants (1) understand the experiences and diverse communication needs of D/deaf and HoH patients, (2) understand the importance of the first contact and apply good practices to reassure D/deaf and HoH patients and promote good follow-up care, and (3) know the different tools available, the basic rules and when and how to use them. Meeting parts of these objectives and consistent with past studies [34, 47], findings showed that participants evinced significant increases in perceived knowledge and self-efficacy in communicating with D/deaf and HoH patients after the intervention. These findings and the fact that increases in both scores remained significant 6 months after the intervention suggest that the intervention may be an effective means to reach sustainable improvements in perceived knowledge of D/deafness, HoH and existing tools to improve communication and in self-confidence in communicating with D/deaf and HoH patients, although future research using more robust design is needed to confirm these findings.

Additional quantitative findings revealed however significant decreases in perceived knowledge and self-efficacy to communicate with D/deaf and HoH patients over time. Consistently, Gilmore and colleagues, who found that medical students attending a workshop on D/deafness had higher score on attitude to D/deafness than their peers who did not attend the intervention, also found a negative association of attitudes to D/deafness with time after completion of the workshop [34]. It may be that beneficial effects of such interventions reduce over time. Hence, as highlighted by our qualitative findings, developing refresher interventions may be necessary to ensure sustainable changes in knowledge and self-efficacy. These refresher interventions might be short, delivered during team meetings or in the form of podcasts.

Additional findings showed that frequency use of basic rules and tools to improve communication as well as colleagues’ remindings of applying the latter did not significantly increase after receiving the intervention. To the best of the authors’ knowledge, this was the first study to assess changes of these organizational payoffs. These findings suggest that alone, the intervention was not sufficient to induce a sustainable change of practices in the working environment. Our qualitative findings suggest a few ways to promote concrete changes of practice in the working environment, including inserting additional practical exercices and benefiting from a resource staff whithin the institution. This resource staff might be an expert or a D/deaf or HoH HCW who would be responsible for providing support to the teams on a day-to-day basis, proposing awareness training for new staff as well as ongoing refreshers for the teams. Combining training and continuous supervision in the field, these resource persons in the field might help to promote sustainable changes in practices, although future research is needed to test this model effectiveness and efficacy.

### Limitations

The results of this study should be interpreted with caution. Firstly, the fact that a majority of participants in each phase of the ressearch project identified themselves as females could represent a bias, even though this prevalence mirrors the higher percentage of females in most professions targeted by the intervention (e.g., nurses, adminsitrative staff). Secondly, whereas the sample in the initial qualitative assessment included 33.3% of physicians, the latter profession was not represented in the intervention test phases. This might indicate a feasibility issue to free half a day to attend the intervention among physicians. Furthermore, inpatient healthcare facilities were not included in the partner institutions. Future research efforts specifically targeting physicians and including inpatient facilities are needed to adapt and evaluate the intervention to these working contexts. Next, the quantitative data used to test the impact of the intervention were derived from self-reported questionnaires, which may be subject to social desirability bias. However, we took steps to mitigate this risk by ensuring the confidentiality of the data. Another limitation pertains to the sample size (*N* = 28; in the test of the finale intervention). In case of small sample (< 30), the GEE sandwich variance estimate may be biased to zero, which increases the risks of type I errors [51]. Although findings from the paired sample t-tests confirmed the findings of the GEE, future larger research is needed to test the intervention effectiveness and efficacy in larger samples.

## Conclusion

To the best of the authors’ knowledge, this was the first quantitative and qualitative longitudinal study involving postgraduate HCWs that evaluated changes on perceived knowledge, self-efficacy in communicating with D/deaf and HoH patients and organizational payoffs after receiving a capacity-building intervention. Main findings revelead that the intervention was followed by significant and sustainable increases in perceived knowledge and self-efficacy in communicating with D/deaf and HoH patients, whereas no significant changes was found for organizational payoffs. Findings of this study open up important perspectives for future research developing and testing a broader intervention aimed at improving communication skills and creating sustainable changes in practice among HCWs and ultimatley improve access and quality of care among D/deaf and HoH individuals.

### Electronic supplementary material

Below is the link to the electronic supplementary material.

Supplementary Material 1

Supplementary Material 2

Supplementary Material 3

Supplementary Material 4

## Data Availability

The datasets is not publicly available due to privacy concerns but may be requested from the corresponding author on reasonable request.

## Abbreviations

HoH: Hard-of-hearing
HCWs: Healthcare workers
IMTEE: Integrated Model of Training Evaluation and Effectiveness
GEE: Population-averaged estimating equation
