# Supplementary material for: Developing a capacity-building intervention for healthcare workers to improve communication skills and awareness of hard of hearing and D/deaf patients: results from a participatory action research study
Source: BMC Health Serv Res. 2024 Mar 6;24:301. doi: 10.1186/s12913-024-10574-3 (PMC10918938; doi:10.1186/s12913-024-10574-3)
Supplement: Supplementary file 1 — Supplementary Material 1 [file 12913_2024_10574_MOESM1_ESM.docx]

**Appendix 1: semi-structured interview grid with D/deaf and HoH patients (phase 1)**

| **Experience with the healthcare system**  **Access**  **Experience**  **Difficulties**  **Adjustments**  **Communication**  **Quality of care** | 1. How does it go when you need to go to the physician or to the hospital?    1. Access (find out where to go, who to contact and how)    2. First contact 2. Once you're in health care setting, how does it go?    1. Difficulties encountered.    2. Adjustments and adaptations, by whom.    3. What helps. 3. What else could help?    1. More time    2. Sign language interpreter, cued speech coder    3. HCWs attitude    4. Communication resources and strategies    5. Adapting to the environment 4. How do you communicate with the HCWs?    1. Difficulties    2. Adaptations    3. Helping or not? 5. What else could help? 6. When communication is poor, what impact does this have on your medical care?    1. Understanding medical indications    2. Risks 7. In general, what do you think of the quality of care you receive when you consult a physician or hospital?    1. Why    2. Examples    3. Possible improvements | *Goal: explore access to and experience with the healthcare system (doctor, hospital)* |
| --- | --- | --- |
| **Facilitating access to the healthcare system** | 1. What would you change in the current healthcare system to better meet your needs?    1. Environment (noise)    2. Other | *The aim is to explore ways of facilitating access to quality healthcare.* |

| **Training** | *As mentioned at the start of our meeting, one of the aims of this project is to develop a training for HCWs and administrative staff.*   1. What do you think they need to know? 2. What could they do better? 3. How should they improve?    1. Differentiating HCWs/administrative staff 4. In your opinion, what form should this training take?    1. Given by whom    2. How to | *Aim: to explore which skills should be targeted by awareness training to meet the specific needs and expectations of the target population.* |
| --- | --- | --- |
| **Closure** | 1. I think I've covered the essential points. What would you like to add? | *Closing question*  *End the interview gently and thank the participant* |
