# Supplementary material for: Developing a capacity-building intervention for healthcare workers to improve communication skills and awareness of hard of hearing and D/deaf patients: results from a participatory action research study
Source: BMC Health Serv Res. 2024 Mar 6;24:301. doi: 10.1186/s12913-024-10574-3 (PMC10918938; doi:10.1186/s12913-024-10574-3)
Supplement: Supplementary file 2 — Supplementary Material 2 [file 12913_2024_10574_MOESM2_ESM.docx]

**Appendix 2: semi-structured interview grid with HCWs and administrative staff (phase 1)**

| **Experience with d/Deaf patients**  **Experience**  **Difficulties**  **Adaptations** | 1. What experience do you have with d/Deaf patients? 2. How often do you meet d/Deaf patients in your working environment? 3. When you meet a d/Deaf patient at work how does it go?    1. Individual experience    2. Perceived experience of the patient    3. Difficulties encountered by the professional    4. Patient's perceived difficulties 4. In general, what helps in this situation*?*     1. Who adapts and how? 5. What strategies do you usually use to adapt to the situation?    1. Strategies for managing communication ?    2. Perceived efficiency? | *Aim: to explore professional experience with d/Deaf patients.*  *(Description, experience, difficulties, how difficulties were overcome, what helped or not)* |
| --- | --- | --- |

| **Experience with HoH patients**  **Experience**  **Difficulties**  **Adaptations** | 1. What experience do you have with HoH patients? 2. How often do you meet HoH in your working environment? 3. When you meet a HoH patient at work how does it go?    1. Personal experience    2. Perceived experience of the patient    3. Difficulties encountered by the professional    4. Patient's perceived difficulties 4. In general, what helps in this situation?    1. Who adapts and how? The patient? The professional (you)? 5. What strategies do you usually use to adapt to the situation?    1. Strategies for managing communication?    2. Perceived efficiency | *Aim: explore professional experience with HoH patients (description, experience, difficulties, how difficulties were overcome).* |
| --- | --- | --- |

| **Intervention**  **Past experience (D/deaf)**  **Expectations and needs**  **Past experience (HoH)**  **Expectations and needs**  **Training content**  **Form** | 1. How do you feel when taking care of a D/deaf patient at work    1. Why 2. What would you need to be able to better welcome/manage a D/deaf patient? 3. What skills would you like to acquire or improve to feel more at ease in this situation? 4. How do you feel when taking care of a HoH patient at work?    1. Why 5. What would you need to be able to better welcome/manage a HoH patients? 6. What skills would you like to acquire or improve to feel more at ease in this situation?   As discussed prior to the interview, we will develop a training for HCWs and administrative.   1. Have you had any training in this area?    1. Location    2. Contents    3. Duration    4. Perceptions 2. What aspects do you think should be covered in this training?    1. Contents    2. Differentiating between the deaf and hard of hearing    3. Objectives 3. In your opinion, what format should this training take?    1. Type (courses, videos or other)    2. Given by whom    3. Location    4. Acceptable duration | *Goals : explore training needs (what it should target and contain)*  *Explore how this training should be organized (format, location, duration, etc.).* |
| --- | --- | --- |

| **Closure** | 1. I think I've covered the essential points. What would you like to add? | *Closing question*  *End the interview gently and thank the participant* |
| --- | --- | --- |
