# Supplementary material for: Developing a capacity-building intervention for healthcare workers to improve communication skills and awareness of hard of hearing and D/deaf patients: results from a participatory action research study
Source: BMC Health Serv Res. 2024 Mar 6;24:301. doi: 10.1186/s12913-024-10574-3 (PMC10918938; doi:10.1186/s12913-024-10574-3)
Supplement: Supplementary file 3 — Supplementary Material 3 [file 12913_2024_10574_MOESM3_ESM.docx]

**Appendix 3: semi-structured interview grid test 1**

| **General perceptions of intervention** | 1. In general, what did you think of the training? 2. What did you like about it?    1. Why?    2. What other aspects did you appreciate? 3. And what didn't you like about it?    1. Why?    2. What other aspects did you dislike? 4. What expectations did you have before participating in the training?    1. To what extent do you feel that the training has met these expectations?    2. What did we miss? 5. What impressed you most during your training?    1. Why? 6. *The objectives of the training were to understand the experiences and communication needs of Deaf and hard-of-hearing people, to understand how to behave with this population, to know the tools available, how and when to use them.*   To what extent do you feel the training meets these objectives?  What did you feel wasn't sufficiently covered?   1. What improvements should be made to the training? | *Goal: to explore general impressions of the intervention and whether it meets the intervention’s aims* |
| --- | --- | --- |

| **Perceptions of training content** | 1. In general, what did you think of the training content? 2. Which parts did you like best?    1. Why 3. Which parts did you like least?    1. Why? 4. What improvements should be made to the training content?    1. What other themes should we add? 5. What do you think of the usefulness of content? | *Goal: to explore impressions of training content and whether these meet expectations and needs in the field; to explore shortcomings; to explore the perceived usefulness of content.* |
| --- | --- | --- |

| **Perceptions of knowledge acquired during training and its application** | 1. What do you remember about the training in general?    1. Aspects improved/strengthened by training? 2. What other skills would you have liked to develop during the training?    1. And what skills?    2. How? 3. How well equipped do you feel to communicate with a d/Deaf or hard-of-hearing patient?    1. Perceived difficulties?    2. How can we overcome them?    3. What to add to the course 4. If you had to explain to your colleagues how to deal with d/Deaf and hard-of-hearing people to improve communication, how would you feel?    1. Training gives you the tools you need?    2. What's more? 5. What changes do you think should be made to facilitate learning during training? | *Aims: explore the perceived impact of training on practice and the perception of acquired knowledge; explore avenues for improvement to facilitate learning.* |
| --- | --- | --- |

| **Organization and form** | 1. What did you think of the training organization? 2. What did you think of the training duration? 3. What did you think of the training general rhythm? 4. How do you feel about the fact it was provided in videoconference? 5. How did you find the speakers?    1. Attitude    2. Clear messages 6. What do you think of the intervention material (slides)? 7. What do you think of the videos? 8. What changes should be made to improve form? | *Goal: to explore impressions of the organization (before and during training). Form, logistics and improvements to be made* |
| --- | --- | --- |
| **Closure** | 1. I think I've covered the essential points. What would you like to add? | *Closing question* |
