# Supplementary material for: Developing a capacity-building intervention for healthcare workers to improve communication skills and awareness of hard of hearing and D/deaf patients: results from a participatory action research study
Source: BMC Health Serv Res. 2024 Mar 6;24:301. doi: 10.1186/s12913-024-10574-3 (PMC10918938; doi:10.1186/s12913-024-10574-3)
Supplement: Supplementary file 4 — Supplementary Material 4 [file 12913_2024_10574_MOESM4_ESM.docx]

**Appendix 4 : semi-structured interview grid test 2**

| **General perceptions of training** | 1. In general, what did you think of the training? 2. What did you like about it? 3. And what didn't you like about it? 4. What expectations did you have before participating to the training?    1. To what extent do you feel that the training has met these expectations?    2. What did we miss? 5. What impressed you most during your training?    1. Why? 6. *The objectives of the training were to understand the experiences and communication needs of d/Deaf and hard-of-hearing patients, to understand what best practices to apply with this population, and to learn about the tools available, how and when to use them.*   To what extent do you feel the training meets these objectives?   1. How useful is the training for your practice?    1. Does it meet your needs?    2. Applicability in your working environment? 2. What improvements should be made to the training? 3. What did you find was lacking? 4. What did you think of the training organization?    1. Duration? Room ? Material ? | *Goal: to explore general impressions of the training and whether it is achieving the objectives set.* |
| --- | --- | --- |

| **Perceptions of knowledge and skills acquired during the training** | 1. What do you remember about the training in general?    1. Experiences and difficulties of deaf and hard-of-hearing people    2. Deaf culture and community    3. The difference between deafness and hard of hearing    4. Diverse needs and means of communication 2. What other skills would you have liked to develop during the course? 3. How well equipped do you feel to communicate with a d/Deaf or hard-of-hearing patient now?    1. Perceived difficulties?    2. How to overcome them (best practices)?    3. What tools could be used? 4. What other skills do you still need to develop? 5. If you had to explain to your colleagues what best practices to adopt with d/Deaf and hard-of-hearing people to improve communication, how would you feel? | *Goal: explore the perceived impact of training on the perception of acquired knowledge and skills* |
| --- | --- | --- |

| **Perception of benefits for the working environment** | 1. In the last 6 months, how did you interact with a person who is deaf or hard of hearing?    1. What tools and strategies, if any, did you use?    2. How did you feel? 2. Now that you've taken the training, what changes do you see in your future practice?    1. Practical strategies and tools to facilitate communication (e.g., interpreter)    2. Teach the teacher (other colleagues) 3. To what extent do you think that using the best practices and tools presented will improve interactions with people who are deaf or hard of hearing? | *Goal: to explore the benefits of training on the practice and experience of professionals from partner institutions.* |
| --- | --- | --- |
| **Closure** | 1. I think I've covered the essential points. What would you like to add? | *Closing question* |
